# Term and definition of a deformity after a spine trauma: Results of an international Delphi study^☆^

**DOI:** 10.1016/j.bas.2024.102749

**Published:** 2024-01-24

**Authors:** E.E.A. De Gendt, S.P.J. Muijs, L.M. Benneker, F.C. Oner

**Affiliations:** a Department of Orthopedics, University Medical Center Utrecht, Utrecht Heidelberglaan 100, 3584 CX, Utrecht, the Netherlands; b Spine Service, Orthopedic Department, Sonnenhofspital, Bern, Switzerland

**Keywords:** Posttraumatic spinal deformity, PSD, Deformity, Impaired function, Spine trauma

## Abstract

**Introduction:**

Deformity of the spinal column after trauma could lead to pain, impaired function, and may sometimes necessitate extensive and high-risk surgery. This ‘condition’ has multiple terms and definitions that are used in research and clinics. A specific term and definition of this condition however is still lacking. A uniform and internationally accepted term and definition are necessary to compare cases and treatments in the future.

**Research question:**

Reach consensus on the term and definition of this deformity after spine trauma using a Delphi approach.

**Material and methods:**

An ‘all-rounds invitation’ Delphi process was used in this study among a group of international experts. The first round consisted of an online survey using input from preparatory studies, a typical clinical case and ICD-11 codes. The second round showed the results in-person and discussion was encouraged. Participants voted for rejection of certain terms. In the third round the final vote took place. When >80 % of the votes was for or against a term the term was rejected or accepted.

**Results:**

Response rate was high (≥84 %). The 3 Delphi rounds were completed. Unanimous voting led to the acceptance of the term and abbreviation as PSD. Deformity in any plane, pain, impaired function, and neurological deficit, were deemed important to include in the definition of PSD.

**Discussion and conclusion:**

Unanimous consensus was reached on ‘Posttraumatic spinal deformity: Condition where a trauma to the spine results in a deformity in any plane and results in pain and an impaired function with or without a neurological deficit.’

## Highlights

• An ‘all-rounds invitation’ Delphi process was used for a term and definition of a deformity after a spine trauma.
• Unanimous agreement was reached on the term Posttraumatic Spinal Deformity (PSD).
• PSD was defined as: Condition where a trauma to the spine results in a deformity in any plane and results in pain and an impaired function with or without a neurological deficit.

## 1 Introduction

It is well known that some deformity after a traumatic injury to the spine is almost inevitable. Many experts have discussed the size of deformity after trauma and the clinical relevance of it to patients (De Gendt et al., 2021; Schoenfeld et al., 2010). However, when and how much deformity after a spine trauma is a problem for patients resulting in pain, impaired function and in some cases necessitating a (re-)operation is still controversial.

The next step to aid in the research and clinical practice of this condition is the development of a uniform and internationally accepted definition, to enable comparability of future research on this topic. Recent research showed that there is still a big discrepancy in opinions on this topic (De Gendt et al., 2023a, 2023b). There is even no agreement on the name of this ‘condition’ as many different names and terms have been used in the literature. Schoenfeld et al. published a definition after sending out a survey to 35 members of the AO Spine community (Schoenfeld et al., 2010). The term they used for this condition was posttraumatic kyphosis and it resulted in this definition: ‘a painful kyphotic deformity after a spine trauma’. This definition is however limited and does not fully depict the condition PSD. The survey was 29-questions long and covered everything from etiology to treatment preferences. This study was however conducted in a time when the participants used different terms for the condition, clouding judgement, and precision of the study. Consequently, the participants did not reach consensus on many factors leading to a limited definition of condition. A systematic review showed that the most common terms used were: ‘spinal posttraumatic deformity’, ‘symptomatic spinal posttraumatic deformity’, ‘symptomatic posttraumatic deformity’, ‘late kyphotic deformity’, ‘chronic vertebral instability’, ‘(severe) posttraumatic kyphosis’, ‘posttraumatic deformity syndrome’, ‘posttraumatic kyphosis’ and ‘spinal deformity, posttraumatic’. (De Gendt et al., 2021).

Many different terms result in many different definitions used in research and clinical practice. The decision making in this condition is challenging because the surgical procedures necessary to correct the deformity are usually very extensive and high risk. The outcome after these procedures is furthermore not always as good as hoped for (D Stoltze J Harms, 2008; El-Sharkawi et al., 2011). Different techniques have been reported, and it is therefore important that comparisons can be made. However, there are no uniform term or definition used for this condition of the spine. This results in the incomparability of treatments of patients with a deformity after a spine trauma.

Next to these arguments, a clear definition can aid in identifying patients with a trauma to the spinal column with risk factors for developing PSD in the future. This could result in better treatment decisions at time of trauma to prevent the development of this condition.

This study aims to provide a universal term and definition of posttraumatic spinal deformity using a Delphi approach in the AO Spine Knowledge Forum Trauma. Clear description of the definition will aid in decision making in clinical practice and aids in accomplishing a uniform language in research of this condition.

## 2 Methods

### 2.1 Study team and participants

A study team was selected to conduct the study. The two members of this team, one researcher (EdG) and one research manager from the AO Spine Knowledge Forum Trauma (KF Trauma), did not take part in de Delphi rounds.

Twenty-four experts from the KF Trauma (orthopedic surgeons and neurosurgeons) with at least 3 years of experience in the treatment of spine trauma were included in this research.

**Fig. 1:**
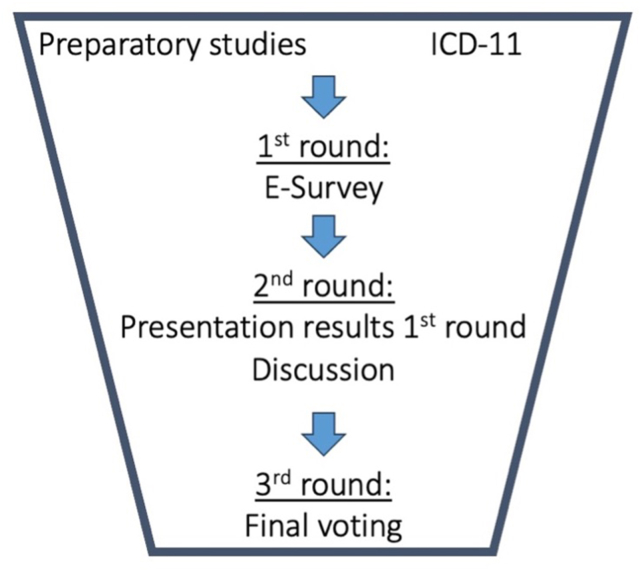
Flowchart of the ‘all-round invitation’ Delphi process to the definition of posttraumatic spinal deformity.

Fig. 1 shows a flowchart of the Delphi process. An ‘all-rounds invitation’ set-up was used for this Delphi study (Boel et al.).

### 2.2 First round: collection of relevant factors

**Fig. 2:**
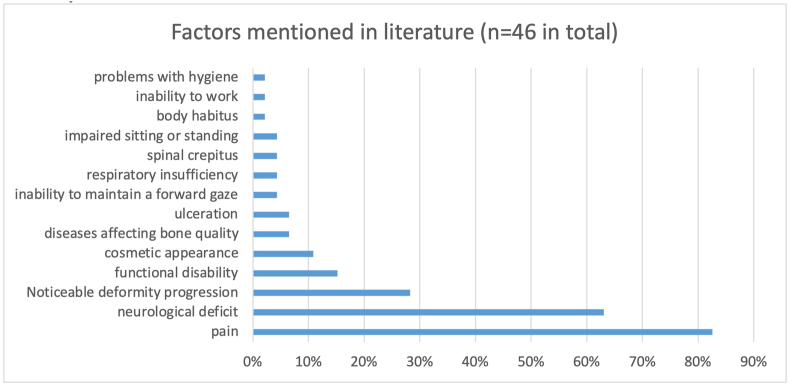
This graph depicts the percentages per factor mentioned in literature on posttraumatic spinal deformity.

First, the information from preparatory studies was collected (De Gendt et al., 2021, 2023a, 2023b). Earlier a preparatory systematic review was performed (De Gendt et al., 2021). This review included 46 articles containing a unique definition or description of a deformity after a spine trauma (Fig. 2). The different terms given to the condition were used as input for the first Delphi round. Additional terms used in other preparatory studies were included (De Gendt et al., 2023a, 2023b). All the terms can be found in Appendix A.

Second, two typical clinical cases were presented to enhance decision making in the survey. Both patients approved the use of their anonymized data including radiological images. After the presentation of the cases the participants were asked to choose which definition they preferred, or if they wanted to add another definition. The participants were asked to justify their choice of definition. The case descriptions including imaging is provided in App1ndix B.

This information was sent to the participants in an online survey comprising of two main questions.

1. If you look at this clinical case, what do you think the condition should be called using a single unifying term (all mentioned terms were collected from the literature through preparatory studies or do you prefer another term)?
2. Please select why you prefer this name? Please check all boxes that apply to you.

Multiple options were given to help in the discussion in round 2.

Third, ICD-11 codes were searched for definitions that were related to spinal or posttraumatic pathology. The terminology and systematics used, were identified, and used in the consensus meeting during the discussion (2nd round).

Fourth, an online survey was sent to all participants. The survey consisted of two questions. The first question was multiple choice and based on the most mentioned factors in the literature. Participants could choose as many as they wanted. The second question was an open field where the participants were asked to describe the condition in their own words.

### 2.3 Second round: discussion

In preparation for the 2nd round the data collected in the first round was analyzed using word clouds and thematic free text analysis. Percentages were given when applicable.

During the second round the results from the first round were presented to the participants at the in-person KF Trauma meeting. Discussion in this round was stimulated by showing the different terms and asking for opinions and why to accept or reject is. Also, example definitions were shown and participants were asked for opinions and reasons to accept or reject certain factors in the definition.

### 2.4 Third round: final voting

In the third round the final voting was done first, per term and second, per factor to be part of the definition. When >80 % of the votes was ‘Yes’ for a factor it was accepted. These factors were than incorporated in a definition which was then voted on until >80 % of the votes was ‘Yes’.

## 3 Results

### 3.1 First round

The first round was started by 19 participants, of which 17 completed the survey. One entry was incomplete, and one participant completed it twice (response rate of 84 %).

#### 3.1.1 Term

Symptomatic spinal posttraumatic deformity was chosen by 53 % of the participants. Four terms were not chosen by any participants: Chronic vertebral instability, (severe) posttraumatic kyphosis, late kyphotic deformity, and spinal deformity, posttraumatic. The results of the entries and percentages of the different terms are stated in Table 1.

**Table 1:** Results of the first Delphi round of the chosen terms.

Possible Index Term	Number of entries	Percentage
Symptomatic spinal posttraumatic deformity	9	53 %
Spinal posttraumatic deformity	3	18 %
Posttraumatic kyphosis	2	12 %
Posttraumatic deformity syndrome	1	6 %
Symptomatic posttraumatic deformity	1	6 %
Other: Symptomatic posttraumatic kyphosis	1	6 %
Chronic vertebral instability	0	
(severe) Posttraumatic kyphosis	0	
Late kyphotic deformity	0	
Spinal deformity, posttraumatic	0	

**Fig. 3:**
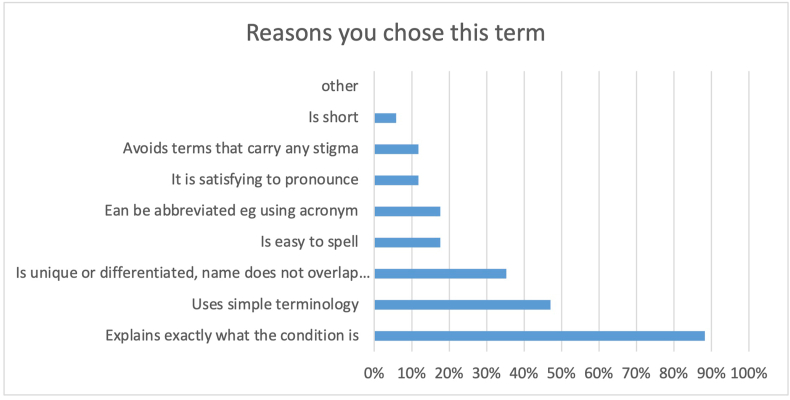
The reasons why participants chose their preferred term given in percentages of total participants (17). Participants could choose more than one.

Almost 90 % of the participants stated that their choice of term ‘explains exactly what the condition is’. In Fig. 3 all the different reasons for choosing the specific terms are depicted.

#### 3.1.2 Definition

**Fig. 4:**
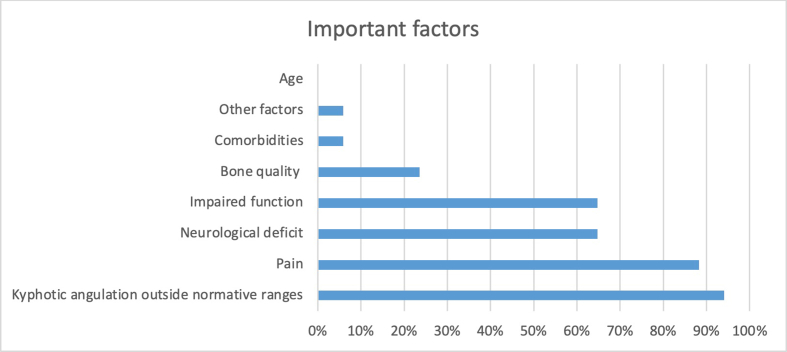
This graph depicts the percentages of respondents that deemed the factors mentioned in literature were important in posttraumatic spinal deformity.

From the systematic review fourteen different factors were extracted. Fig. 4 shows the different factors mentioned in the articles as percentages from the total number of articles.

Two similar definitions were extracted from the ICD-11 codes (Appendix A). The systematics used in the ICD-11 definitions focusses on the pathology, the symptoms, or the cause of the condition/term. This systematics was explained and discussed during the second round and helped structure the final definition in the third round.

**Fig. 5:**
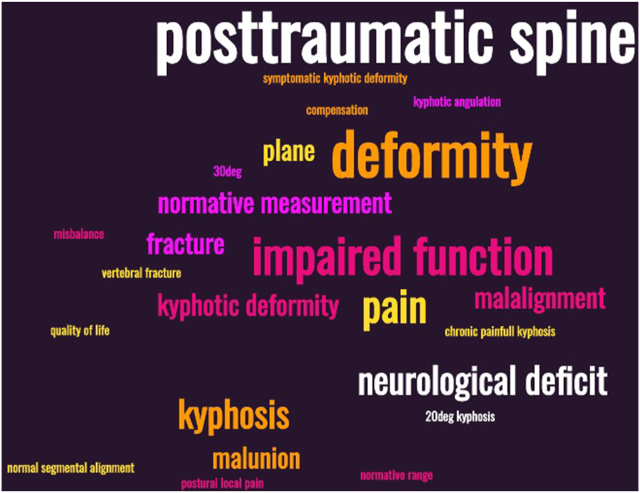
The magnitude of the factors is directly related to the number of times that factor was mentioned by participants.

In total seventeen participants completed the online survey (response rate of 84 %), one completed it twice and one entry was incomplete. The results of the first question are presented in Fig. 3. The descriptions of PSD from the second question were processed and presented to the participants as seen in Fig. 5.

### 3.2 Second round

All the results from the first round were presented to the participants during the in-person meeting (24 participants were present). Personal and common reasons to include or exclude certain parts of the term and factors of the definition were discussed. All 24 participants voted, none refrained.

#### 3.2.1 Term

The mentioning of ‘kyphosis’ in the term was discussed as it excluded deformities in other planes that are deemed possible in this condition. The word ‘symptomatic’ was discussed, with supporters and opponents. Also, the following terms with the least or no votes in the first round were discussed and rejected by >80 % of the participants: Posttraumatic deformity syndrome, Symptomatic posttraumatic deformity, Other: Symptomatic posttraumatic kyphosis, Chronic vertebral instability, (severe) posttraumatic kyphosis, Late kyphotic deformity and Spinal deformity, posttraumatic. This resulted in the following terms that were included for voting in the third round: Symptomatic spinal posttraumatic deformity, Spinal posttraumatic deformity, and Posttraumatic kyphosis.

#### 3.2.2 Definition

There was discussion on the word ‘kyphosis’, it was stated that also deformity in other planes could be present and that those should not be excluded in the final definition.

Another topic of discussion was whether to add a certain timeframe to the definition. It was decided that this was not applicable because it would limit the possible inclusion of certain patients in the future. The factor ‘neurological deficit’ was discussed as being not always present but still an important part in this condition and therefore that it should be included in the definition without excluding any patients. It was proposed to add a certain cut-off value of an angular measurement, from the results of the survey >20 or >30° were mentioned by two separate participants. The discussion concluded that no such cut-off value could be added because no consensus existed in current literature or amongst experts.

The following example definitions were presented and discussed: ‘Condition where a trauma to the spine results in.

- a deformity in any plane of the spine (outside normative ranges) and results in pain, an impaired function and can be accompanied with a (increasing) neurological deficit.
- a malalignment of the spine in any plane, pain, and impaired function.’

### 3.3 Third round

#### 3.3.1 Term

A definitive voting process was started in which the research team posed the terms. In the end a unanimous agreement was reached for the term Spinal Posttraumatic Deformity. No one refrained from voting. Also, with several native English participants present it was decided that the order of the words should be adjusted. And the term: Posttraumatic Spinal Deformity with PSD as abbreviation was unanimously accepted by the participants.

#### 3.3.2 Definition

After the discussion of the second round, >80 % of the participants voted to include the factors: pain, impaired function, deformity in any plane, and neurological deficit in the definition. Using the ICD-11 code systematics a final definition was proposed and voted upon. The final definition of posttraumatic spinal deformity: *‘Condition where a trauma to the spine results in a deformity in any plane and results in pain and an impaired function with or without a neurological deficit.’*

This definition was unanimously accepted as the new definition of posttraumatic spinal deformity.

## 4 Conclusion

When the name and definition of a condition is not unanimously accepted, comparison in treatment, research and outcomes can be difficult. This study focused on the creation of the term and the definition of the condition: a deformity after a spine trauma, through an ‘all-rounds invitation’ Delphi process. The participants unanimously accepted *posttraumatic spinal deformity (PSD)* with the definition: ‘Condition where a trauma to the spine results in a deformity in any plane and results in pain and an impaired function with or without a neurological deficit.’

## 5 Discussion

A Delphi study is an ideal format to explore all the different opinions and discuss them in a group of experts. We decided to use an adjusted format of the Delphi study, using preparatory studies as well as an online survey in preparation. This decreased time spent for the participants and enhanced the response rate. We did think it important to have in-person discussion to enhance the quality and acceptance of the new definition. Those who were not able to be present in-person were able to attend through video connection.

An ‘all-rounds invitation’ was used in this Delphi study. The original set-up of a Delphi study uses only respondents of previous rounds for consecutive rounds. An ‘all-rounds invitation’ set-up allows participants to join in the consecutive round regardless of their earlier participation. In 2021, Boel et al. researched the difference between the two approaches in an e-Delphi study (Boel et al.). They found a lower overall response rate for the original set-up (46 %) compared to the ‘all-rounds invitation’ (61 %). No differences were found in the percentages of critical votes or consensus results. Concluding that an ‘all-rounds invitation’ approach is not inferior to the original set-up and might be beneficial. Our study found a high (17 out of 19 participants) response rate in the first round. And a complete response rate in the second and third round. The second and third round were during the in-person meeting and discussion. There was a possibility of refraining from voting, but no participant chose to refrain from voting.

Schoenfeld et al. did great work trying to define this condition (Schoenfeld et al., 2010). But with an absent uniform defined and specified term this task was difficult, the term Schoenfeld et al. choose was: posttraumatic kyphosis. This excludes a group of patients who do suffer from PSD, and have a deformity in another plane of the spine. Also, just one big survey among the experts was not enough to get all different views and experiences on the same page. They reported great variance in opinions and were not able to add other factors to their definition.

A limitation of this study was mostly in the wording of the typical clinical case. It had to be without any implications or judgements and in correct and clear wording. Another limitation came up during one of the discussions in the last round. An adequate abbreviation should be decided because this would limit confusion when the term will be used in future research. An adequate abbreviation of the term ‘posttraumatic spinal deformity’ could be PTSD, but this is already a widely accepted abbreviation for posttraumatic stress disorder. It was then decided by unanimous voting that PSD was an adequate definition without big associations to other conditions.

In the future our aims are to submit the term posttraumatic spinal deformity and the definition to be included in the ICD-11 codes. When a term and definition are included in the ICD-11 codes, they are internationally accepted and findable for a major public. This will aid in the increased awareness of the condition posttraumatic spinal deformity in research and clinical practices.

In the future we would like to progress this research by setting up an international cohort study where patients after a spine trauma are followed using the AO Spine PROST up to ten years with additional radiographs to measure progression or stability. The idea is that we can then see which patients yield a high risk to develop PSD and see which factors they have in common. If those factors are present in a patient who arrives at the hospital after a trauma to the spinal column, we could decide to treat that patient, accordingly, depending on the presence or absence of risk factors for developing posttraumatic spinal deformity in the future.

## Funding statement

No funding was received for this specific study.

## Declaration of interests

The authors declare the following financial interests/personal relationships which may be considered as potential competing interests:Editorial Capacity of FC Oner in special issue If there are other authors, they declare that they have no known competing financial interests or personal relationships that could have appeared to influence the work reported in this paper.

## Figures and Tables

**Table:** ICD-11 codes

ICD-11 code	Term	Definition
FA70.0	Kyphosis	This is a curving of the spine that causes a bowing or rounding of the back, which leads to a hunchback or slouching posture.
ME 84	Spinal Pain	This is a condition characterised by pain felt in the back that usually originates from the muscles, nerves, bones, joints or other structures in the spine.

**Figure:**
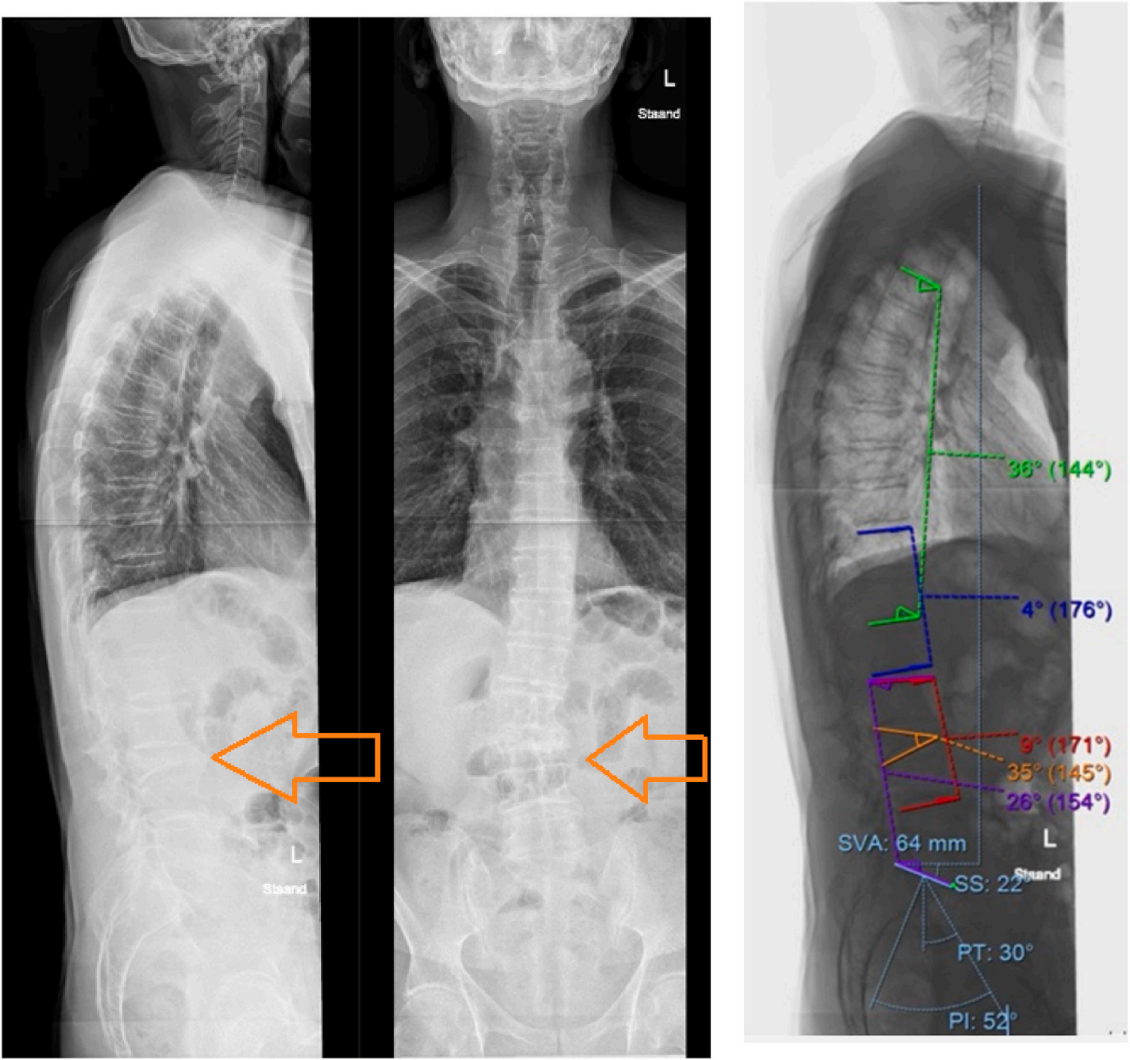


**Figure:**
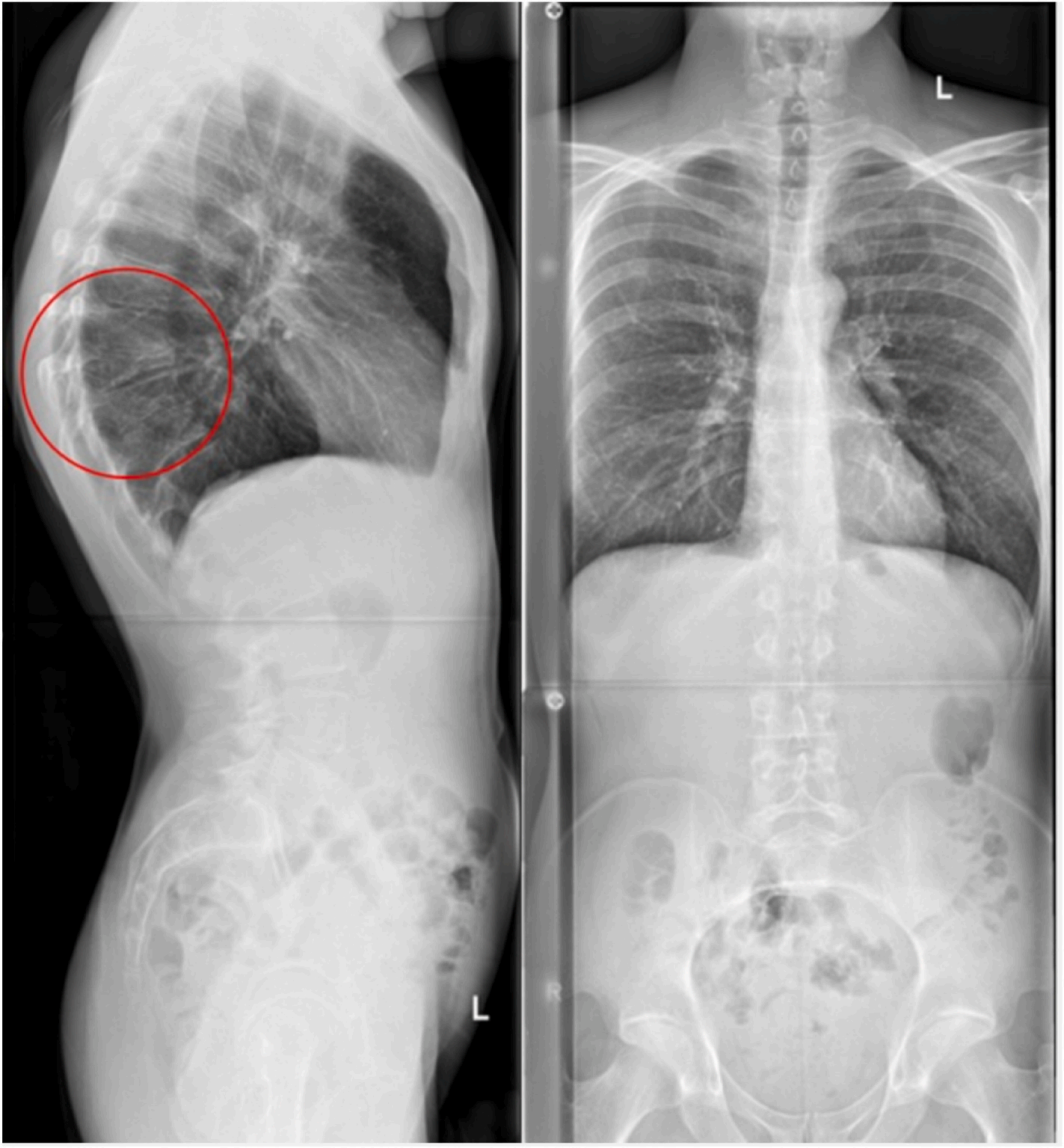


## APPENDIX A

Chronic vertebral instability.

Late kyphotic deformity.

Posttraumatic deformity syndrome.

Posttraumatic kyphosis.

(severe) Posttraumatic kyphosis.

Spinal deformity, posttraumatic.

Spinal posttraumatic deformity.

Symptomatic posttraumatic deformity.

Symptomatic spinal posttraumatic deformity.

## APPENDIX B

### Case description 1

A male of 64 years presents himself at the outpatient clinic. Two years ago he fell from a ladder of about 2,5 m height. He suffered from a L3 A4 fracture and was treated with a Hewitt brace for 6 weeks.

He complains of increasing pain in his lower back over the last year and has increasing problems with lifting his upper right leg. He does not suffer from urine or defecation problems. There are no other neurological complaints.

Physical examination shows an absent lumbar lordosis and a hyperkyphotic spine.

Neurological examination shows: MRC5- of the left quadriceps also pain related and no other neurological deficits.

### Case description 2

A male of 35 years presents himself at the outpatient clinic. He suffered from a T9 B2 fracture and T10-11-L1 A1 type fractures. He was treated with 6 weeks of bed rest.

Patient now complaints of increasing pain in the lower back and more arching forward of his upper back. Physical examination shows a hyperlordosis with maximum pelvic rotation.
